# Factors influencing cancer genetic somatic mutation test ordering by cancer physician

**DOI:** 10.1186/s12967-020-02610-7

**Published:** 2020-11-12

**Authors:** Anastassia Demeshko, David J. Pennisi, Sushil Narayan, Stacy W. Gray, Matthew A. Brown, Aideen M. McInerney-Leo

**Affiliations:** 1 The Dermatology Research Group, University of Queensland Diamantina Institute, The University of Queensland, Translational Research Institute, 37 Kent St, Woolloongabba, QLD 4102 Australia; 2 Translational Genomics Group, Institute of Health and Biomedical Innovation, School of Biomedical Sciences, Queensland University of Technology (QUT), Translational Research Institute, 37 Kent St, Woolloongabba, QLD 4102 Australia; 3 Department of Population Science, City of Hope, Duarte, CA USA; 4 Department of Medical Oncology & Therapeutics Research, City of Hope, Duarte, CA USA; 5 Guy’s and St Thomas’ NHS Foundation Trust and King’s College London NIHR Biomedical Research Centre, London, UK

**Keywords:** Cancer physicians, Somatic, Attitudes, Genomic, Ordering, Confidence

## Abstract

**Background:**

Clinical whole exome sequencing was introduced in an Australian centre in 2017, as an alternative to Sanger sequencing. We aimed to identify predictors of cancer physicians’ somatic mutation test ordering behaviour.

**Methods:**

A validated instrument assessed somatic mutation test ordering, genomic confidence, perceived utility of tumour molecular profiling, and percent of patients eligible for targeted therapy. A cash incentive was included in 189/244 questionnaires which were mailed to all Queensland cancer specialists in November 2018.

**Results:**

110 participated (response rate 45%); 54.7% oncologists, and the remainder were surgeons, haematologists and pulmonologists. Oncologists were more likely to respond (p = 0.008), and cash incentive improved the response rate (p < 0.001). 67/102 (65.7%) of physicians ordered ≥ 5 somatic mutation tests annually. Oncologists saw 86.75 unique patients monthly and ordered 2.33 somatic mutation tests (2.2%). An average of 51/110 (46.1%) reported having little/no genomic confidence. Logistic regression identified two significant predictors of somatic mutation test ordering: being an oncologist (OR 3.557, CI 1.338–9.456; p = 0.011) and having greater confidence in interpreting somatic results (OR 5.926, CI 2.230–15.74; p < 0.0001).

**Conclusions:**

Consistent with previous studies, the majority of cancer physicians ordered somatic mutation tests. However, the percentage of patients on whom tests were ordered was low. Almost half respondents reported low genomic confidence. Somatic mutation test ordering was higher amongst oncologists and those with increased confidence in interpreting somatic variants. It is unclear whether genomically confident individuals ordered more tests or whether ordering more tests increased genomic confidence. Educational interventions could improve confidence and enhance test ordering behaviour.

## Background

In both solid and haematological malignancies, the understanding of how cancer genomic profiles influence treatment responses and outcomes is rapidly increasing. This increased knowledge, combined with the falling costs and increasing availability of next-generation sequencing (e.g. gene panels, whole-exome (WES) and whole-genome sequencing (WGS), is driving increased use of cancer somatic mutational profiling in clinical practice [1]. Cancer somatic mutational profiling is now a recommended component of the workup of many common cancer types, and has also been shown to improve outcomes in some cancers subtypes following failure of initial standard therapy [2].

Critical to the uptake of this new methodology is expertise and confidence amongst oncologists. Little is known about physicians’ knowledge, attitudes and utilisation of genomic testing. Physicians’ willingness to incorporate genetic testing into clinical practice is likely to be influenced by factors related to the testing itself such as its availability, cost and clinical performance. In addition, it may be influenced by physician-related factors such as perceptions regarding utility and consequences of testing, comfort and confidence in dealing with genetic testing, and level of education, training and experience in regards cancer genomics [3, 4]. The Capability, Opportunity and Motivation Model of Behaviour (COM-B) is a framework for identifying existing barriers and facilitators to the adoption of new medical interventions, such as genetic testing [5]. This theory posits that the interaction of capability, opportunity, and motivation modify behaviour [3].

US studies have shown that cancer physicians have low levels of comfort and personal confidence with genomics [6–8]. An international study of breast cancer specialists found that the 38% who routinely ordered tests had high confidence in their genomic knowledge [9]. A single Australian study explored cancer physicians’ attitudes towards mainstreaming *BRCA1/2* testing to inform treatment choices [10]. The 36 respondents perceived that treatment-focused genetic testing was useful in informing management. Confidence was not assessed.

Whilst large multi-gene screening panels and WES/WGS have been available in the United States for almost a decade [2], and is available in the United Kingdom through the National Health Service, this testing has been available for cancer (clinical/research) in Australia for < 5 years. Following the introduction of clinical WES on paired tumour-normal cancer samples sequencing within Metro South Hospital and Health Service, and its availability throughout Queensland through Pathology Queensland, from January 2017, we administered a questionnaire to evaluate which COM-B elements were associated with somatic mutation test ordering behaviour amongst cancer physicians.

## Methods

### Study population and ethics

This study was ethically approved by Metro South Hospital and Health Service (MSHHS)(HREC/17/QPAH/225) and Queensland University of Technology Human Research Ethics Committees. A database was created using publicly available sources to include all specialist physicians (oncologists, haematologist, pulmonologists, surgeons etc.) known to be providing specialist cancer management to adult cancer patients in Queensland. Dermatologists were excluded as tumour molecular profiling is less relevant in non-melanoma skin cancer. This survey was administered in November 2018.

To ascertain whether an incentive improved response rate, AUD$10 cash was randomly included in 75% of surveys and the remainder did not receive any incentive.

### Somatic mutation testing service

Clinical testing at the Metro South public hospital is performed by the Australian Translational Genomics Centre, accredited by the National Association of Testing Authorities. Whole exome sequencing, plus a high coverage “spike in” panel of 523 cancer-associated genes, is performed on both tumour and germline samples. Variants are classified according to the CIVIC system [11]. Two reports are generated on each patient. Both reports include all actionable, somatic and germline A, B and C variants. The first is specific to the primary diagnosis and the second is a pan-cancer report. In both reports, variants are classified depending on whether they inform diagnosis, prognosis or treatment and links are provided to supporting evidence. Between 1 and 5 variants are identified per patient. Samples of both test reports are now included in Additional file.

### Survey instrument and study procedures

We adapted a previously developed and validated instrument [8] to include an additional question and customise demographics. The final 20-item instrument included both standard and novel measures (Additional file 1). Seven Likert items assessed physicians’ attitudes regarding disclosure of genomic sequencing results depending on actionability. Specifically, Tier 1 results were clinically actionable, validated and/or FDA-approved, Tier 2 were potentially actionable, and Tier 3 encompassed all other genomic variants. An additional three items evaluated physicians’ confidence regarding somatic variants: interpreting, explaining, and making treatment recommendations. One item assessed physicians’ confidence in identifying genomics experts. Three items assessed current cancer, somatic, germline and pharmacogenomics test ordering behaviour. One item captured the percentage of patients for whome molecularly indicated agents were thought to be available/accessible. One newly added item, a visual analogue scale, asked physicians to rate the extent to which tumour pathology informed treatment choices as compared to tumour molecular profile (TMP). Three sociodemographic questions captured specialty (customised to target population), years’ post-graduation and number of unique patients per month. A final open-ended question captured additional feedback.

### Data and statistical analyses

Participant characteristic were summarised. All analyses were performed on *SPSS Statistics* and/or *GraphPad Prism 7.0.* Descriptive analyses were conducted for demographic variables, number of patients, and attitudes towards the actionability and relative utility of TMP. Chi squared tests were used to evaluate the likelihood of disclosing Tier 1, 2 and 3 results were compared, and the relative frequency of ordering different genetic test types. Stepwise backward logistic regression analysis was performed to identify which variables predicted somatic mutation test ordering behaviour. These included: years’ experience (1–20/> 20 years); specialty (oncologist/non-oncologist); number of unique patients per month (0–20/> 20 patients); perceived value of TMP relative to pathology; confidence (low/high) in interpreting and making treatment recommendations based on somatic mutation tests. For each association, the odds ratio (OR) and 95% confidence interval (CI) was calculated. Results were considered significant when p < 0.05.

## Results

### Descriptive statistics

Of the 244 physicians surveyed, 110 completed the questionnaire and 20 returned surveys, declining participation. The overall response rate was 45% and was higher in the cash incentive (n = 99/189, 52.4%) as compared no incentive groups (n = 11/55, 20%; p < 0.0001). Oncologists were more likely to respond (58/106, 54.7%) compared to non-oncologists (51/138, 36.9%; p = 0.008). There was a similar distribution of specialties in the cash incentive and no incentive groups but the latter group was too small (n = 11) to allow analysis. 101/110 (91.8%) completed all questions. The population characteristics and survey responses are summarised in Table 1.

**Table 1:** Participant characteristics (n = 110)

Characteristic	Participants
Years since medical school, No. (%)	
0–10	4 (3.6)
11–20	43 (39.1)
21–30	43 (39.1)
> 30	15 (13.6)
Not completed	5 (4.6)
Physicians caring for cancer patients, %	
Oncologist	58 (52.7)
Non-oncologist (haematologist, pulmonologists, surgeons etc.)	51 (46.7)
No. of unique patients per annum (based on monthly estimates)	
All physicians (Mean)	1196.4
Oncologists (Mean)	1041.0
Availability/Accessibility of molecularly indicated agents,%^a^	
Mean	39.13
Extent to which tumour pathology informs treatment choices as compared to tumour molecular profile, cm^b^	
Mean	4.06
Median	3.60
Low levels of confidence relating to somatic genomic results, No. (%)	
Interpreting	56 (50.9)
Explaining	44 (40.0)
Making treatment recommendations	52 (47.3)
Mean	50.7 (46.1)
Low levels of confidence in identifying consultants	
No. (%)	31 (28.2)

^a^ Due to a printing error, responses could be analysed in only 67 cases

^b^ Measured on a 10 cm line, with tumour pathology on the left and tumour molecular profile on the right

On a 10 cm line, with tumour pathology on the left and TMP on the right, the mean was 4.1 cm and the median was 3.7 cm. 51/104 (49.0%) respondents marked 3.6 cm or less, indicating that TMP factored little, if at all, in their treatment decision making.

An average of 51 of physicians (46.1%) reported low genomic confidence in all three domains (interpreting, explaining and making treatment recommendations based on somatic results) whereas only 31/110 (28.2%) reported low levels of confidence in identifying genomic consultants. Physicians were significantly more likely to be confident in identifying genomics consultants than they were in interpreting, explaining or making treatment recommendations based on somatic results themselves (p < 0.0001).

### Test ordering

65.7% of cancer physicians ordered ≥ 5 somatic mutation tests per year, which is significantly higher than the 48.1% ordering ≥ 5 germline cancer tests (p < 0.009) and the 11% ordering ≥ 5 cancer pharmacogenomic tests (p < 0.00001)(Table 2). The annual mean number of somatic mutation tests, in responders, was 27.4 or 25.8 in oncologists. The average number of unique patients seen by oncologists was 1041 (86.75/month × 12). Thus, 25.8/1041 (2.5%) of cancer patients were offered somatic mutation testing.

**Table 2:** Chi-squared analysis of cancer somatic, germline and pharmacogenomic test ordering behaviour in the past year

	No. (%) of physicians ordered zero tests	No. (%) of physicians ordered ≥ 5 tests/year	Mean	Median	Range	p-value*
Somatic mutation tests *(n *= *102)*	17 (16.7%)	67 (65.7%)	27.4	10	0–200	
Germline tests *(n *= *102)*	39 (38.2%)	48 (48.1%)	7.2	2	0–50	0.009**
Cancer Pharmacogenomic tests *(n *= *100)*	75 (75%)	11 (11%)	1.04	0	0–20	< 0.00001**

*p-value based on Chi squared analysis of proportion who ordered < 5 and ≥ 5 tests per year

**Compared to somatic mutation test ordering

Likelihood of disclosing different tiers of somatic mutation test results are summarised in Table 3. Participants were more likely to disclose Tier 1 or Tier 2 results as compared to Tier 3 (p < 0.0001). There was a preference for disclosing Tier 2 results which conferred eligibility to a clinical trial as compared to Tier 2 results which required off-label use of a previously approved drug (p < 0.0001). There was a trend to disclose prognostically favourable Tier 2 variants, compared to prognostically unfavourable results (p = 0.06).

**Table 3:** Chi-squared analysis of attitudes towards the disclosure of somatic mutation test results

	Likely to disclose	Likely to not disclose	*p*-value
Tier 1 and 2 vs Tier 3			
Tier 1 and 2 (average)	83.3	17.2	< 0.0001*
Tier 3	27	62	
Tier 2 prognosis			
Favourable prognosis	94	7	0.0632
Unfavourable prognosis	84	15	
Tier 2 treatment			
Phase II clinical trial	98	5	< 0.0001*
Off label	70	30	

*p < 0.05

Stepwise backward logistic regression analysis (see Table 4) showed that oncologists, and those with greater confidence in interpreting genomic test results, were more likely to order somatic mutation tests (OR 3.6, p = 0.011 and OR 5.9, p < 0.0001, respectively).

**Table 4:** Backward stepwise logistic regression model for survey results identifying the predictors of somatic mutation test ordering

Predictor variable	No. of somatic mutation tests ordered			
	Sig.	OR	95% CI for OR	
			Lower	Upper
Step 1				
Years of experience	0.797	1.07	0.62	1.86
Specialty	**0.013**	**3.52**	**1.30**	**9.53**
Unique number of patients	0.733	0.95	0.68	1.31
TMP vs TP^a^	0.819	0.98	0.80	1.19
Confidence in interpreting	**0.072**	**3.14**	**0.91**	**10.87**
Confidence in making treatment recommendations	0.126	2.48	0.77	7.96
Step 2				
Years of experience	0.793	1.08	0.62	1.86
Specialty	**0.013**	**3.55**	**1.31**	**9.60**
Unique number of patients	0.744	0.95	0.69	1.31
Confidence in interpreting	**0.062**	**3.21**	**0.94**	**10.93**
Confidence in making treatment recommendations	0.124	2.49	0.78	7.97
Step 3				
Specialty	**0.013**	**3.53**	**1.31**	**9.52**
Unique number of patients	0.726	0.94	0.69	1.30
Confidence in interpreting	**0.055**	**3.28**	**0.97**	**11.08**
Confidence in making treatment recommendations	0.130	2.42	0.77	7.63
Step 4				
Specialty	**0.012**	**3.57**	**1.33**	**9.61**
Confidence in interpreting	**0.045**	**3.41**	**1.03**	**11.32**
Confidence in making treatment recommendations	0.132	2.41	0.77	7.60
Step 5				
Specialty	**0.011**	**3.56**	**1.34**	**9.46**
Confidence in interpreting	**0.000**	**5.93**	**2.23**	**15.75**

Bold denoted *p* < 0.05

^a^ Measured on a 10 cm line, with tumour pathology on the left and tumour molecular profile on the right

### Qualitative comments

Twenty-three individuals provided comments to an open-ended question, and a further ten spontaneously added written comments beside specific questions. Qualitative analysis identified five common themes: test costs and reliability concerns, lack of physician education, lack of infrastructure to support the testing and counselling of patients, the utility of sequencing was cancer type dependent, and the recognised potential for genomics to improve cancer therapies. Illustrative quotes can be found in Additional file 1: Table S1.

## Discussion

This is the first Australian study to focus on factors predicting somatic mutation test ordering amongst cancer physicians. Oncologists were more likely to respond than non-oncologists and cash incentive improved overall response rate. The response rate (RR) in this study was consistent with United States physician surveys where questionnaires without incentives have a response rate of < 30% as compared to ≥ 50% when incentives are included [12]. Whilst cash incentives have been shown to increase the RR among North American clinicians [13], and Australian pharmacists [14], to our knowledge, this is the first study to report similar findings in Australian cancer specialists.

Physicians order more somatic mutation tests than either cancer germline or pharmacogenomic tests with two-thirds ordering at ≥ 5 somatic tests in the past year. This is similar to a large study of United States colorectal and thoracic oncologist survey where 31–68% ordered ≥ 5 somatic mutation tests annually [15]. That study evaluated test ordering for specific somatic variants and found that ordering rates varied significantly depending on the variant, and whether there were endorsed guidelines. Overall, fewer than 3% of Queensland cancer patients were offered somatic mutation testing, which is lower than reported in one sub-specialty study. Specifically, 13% (20/153) of lung or colorectal cancer patients from a small group of United States thoracic and gastrointestinal oncologists (n = 27) [16]. In contrast, an international study of sequencing behaviours in breast cancer specialists found that only 38% ordered any somatic mutation testing [9], which is lower than the 83.3% of Queensland specialists. Furthermore, within the cohort of specialists who did order tests, 68% reporting doing so on ≤ 5% of their patients, which more closely reflects our findings. Physicians’ qualitative comments, in our study, suggest that the low uptake may be, in part, attributable to uncertainty regarding the actionability of the results, which is consistent with qualitative data reported previously [16]. However, one question in the present study specifically assessed physicians’ perceptions on the percentage of patients who would have access to molecularly indicated agents, and the mean was 40%. This is considerably higher than the somatic mutation test ordering rate would imply.

The median germline testing was similar to previous reports with thoracic and gastrointestinal oncologists also ordering a median of two tests per annum [16]. In fact, physicians in this study ordered a mean of seven germline tests in the preceding 12 months, which is higher than the average of three ordered by United States gastrointestinal oncologists [15]. Similarly, in this study, a quarter of cancer physicians reported ordering pharmacogenomic tests which is consistent with a study which found that 35% of lung and colorectal oncologists ordered at least one pharmacogenomic test in the previous year [16].

In order to more fully understand attitudes towards genomic testing, clinicians were asked to rate the relative utility of tumour pathology compared to TMP in informing treatment decisions. They were not equally valued, as initially predicted; rather, tumour pathology was weighted more heavily. Qualitative comments stated that the relative value of TMP is dependent on the tumour type, as has been clinically proven [2]. To our knowledge, this is first study to evaluate the perceived, relative utility of each. It would be meaningful to readminister the survey in 5 years’ time to capture any change over time.

Cancer physicians were, understandably, more inclined to disclose Tier 1 or Tier 2 results than Tier 3. Consistent with our results, previous research has shown general support for the disclosure of Tier 1 and 2 results [8, 16]. Furthermore, in parallel to our findings, United States physicians were more likely to report a Tier 2 result which conferred eligibility to a Phase II clinical trial than to disclose a Tier 2 variant associated with off-label drug use [16]. However, less than a quarter of respondents in this study supported disclosing Tier 3 results (disclosing as many sequencing results as the patient wanted, including raw sequencing data) as compared to 50–96% in previous studies [8, 16]. Qualitative comments from one study clarified that willingness to consider disclosure of Tier 3 variants stemmed from the desire to optimise treatment choices [15].

Almost half of physicians reported low to negligible confidence in interpreting, explaining and making treatment recommendations relating to somatic TMP results (‘genomic confidence’). Although oncologists have greater confidence in interpreting variants than other cancer specialists, a substantial portion still report low levels of genomic confidence, consistent with previous medical oncology studies [8, 9, 16, 17]. Overall, the portion reporting low confidence in this study is considerably higher than the ~ 20% of cancer physicians internationally [8, 9]. This is significant as research has shown that low confidence negatively affects clinical practice and ordering behaviours [18]. Of relevance, a systematic review article exploring the integration of genetics into healthcare found a lack of access to genetics services was a significant barrier [19]. In this study, the majority reported confidence in their ability to identify genetics consultants, suggesting that this is less of a barrier in Queensland.

In regression modelling, the only two predictors of somatic mutation test ordering were being an oncologist, and having greater confidence in interpreting somatic variants. Previous research has shown that oncologists are more likely to order genetic testing than other cancer specialists [15, 20], possibly offering insight into their increased likelihood to participate in the study in the first place. Genomic confidence generally, has been associated with a greater intention to request genetic tests [8] and a higher uptake in practice [20, 21]. However, in this study, it is unclear whether confidence predicts ordering behaviour or whether those who order more tests become more confident in interpreting results.

Under the COM-B theory [5], test ordering behaviour should be predicted by a combination of Capability, Opportunity and Motivation, and the results of this study are consistent with this theory. Specifically, specialty (capability), and greater confidence in interpreting results (motivation) are associated with ordering behaviour. Importantly, it has been repeatedly shown that capability and motivation can be moderated through education as evidenced by increases in confidence and perceived competence [22–24]. Furthermore, educational interventions have been shown to increase genomic confidence amongst cancer specialists [25]. Of relevance to this program, there is a growing body of evidence that face-to-face training positively affects attitudes and behaviors, and is arguably most effective when provided “just in time” [26]. Therefore, these findings imply that introducing targeted educational programs, which increase capability and motivation, immediately prior to offering new tests, could modify physicians’ somatic ordering behaviours.

The limitations of this study include a 52% response rate, capturing just 58% of oncologists, so results are not representative of all Queensland cancer specialists. In addition, this is a heterogeneous sample and the practices of the oncologists will not usually be consistent with the practices of non-oncologists. Of note, this survey assessed physicians’ perceptions of ordering behaviour rather than their actual somatic mutation testing rate. It is possible that the actual ordering behaviour was higher. A printing error for the question assessing actionability means that only 67 responses to this question could be analysed.

## Conclusion

This study demonstrated that the majority of Queensland cancer specialists are ordering somatic mutation tests, but suggests that less than three percent of patients are offered testing. Test ordering was associated with greater confidence in test interpretation and being an oncologist, both of which can be enhanced through educational interventions. This has implications for the training of existing cancer specialists and medical students.

## Supplementary information

**Additional file 1.** Questionnaire and Qualitative Comments

## Data Availability

The datasets for this article are not publicly available due to concerns regarding participant/patient anonymity. Requests to access the datasets should be directed to the corresponding author.

## Abbreviations

CI: Confidence interval
COM-B: Capability, Opportunity and Motivation Model of Behaviour
OR: Odds ratio
RR: Response rate
TMP: Tumour molecular profile
WES: Whole exome sequencing
WGS: Whole genome sequencing
