# Supplementary material for: Factors influencing cancer genetic somatic mutation test ordering by cancer physician
Source: J Transl Med. 2020 Nov 12;18:431. doi: 10.1186/s12967-020-02610-7 (PMC7663861; doi:10.1186/s12967-020-02610-7)
Supplement: Supplementary file 1 — Additional file 1. Questionnaire and Qualitative Comments [file 12967_2020_2610_MOESM1_ESM.pdf]

Supplemental Data

Questionnaire

Please indicate the extent to which you agree or disagree with the following statements about the return of genomic sequencing information to patients:

	Strongly disagree	Somewhat disagree	Neither agree nor disagree	Somewhat agree	Strongly agree
1. Patients should *only* be offered their genomic sequence results if evidence demonstrates that actions based on the results can change patient management decisions and improve net health outcomes.	☐	☐	☐	☐	☐
2. Patients should be offered genomic sequence results for which there is an *established relationship between genotype and phenotype* (e.g., results can be used to diagnose a disorder or to assess risk for a disease), even if the results do not alter management decisions or improve net health outcomes.	☐	☐	☐	☐	☐
3. Patients should be *offered as many of their genomic sequence results as they want*, up to and including their raw genomic sequence data.	☐	☐	☐	☐	☐

The questions that follow contain scenarios describing a particular type of genomic alteration derived from sequencing the patient's tumour DNA. **Assume:**

- the tumour DNA belongs to YOUR adult patient with a metastatic solid tumour
- the sequencing was performed in a NATA certified lab
- the patient is currently receiving a first-line standard chemotherapy regimen
- the patient is active and capable of self-care
- the patient has indicated that s/he would like to be told about all clinically valid genomic results

Please check the box that reflects how likely you would be to disclose the information described in each scenario to your patient.

Please read each scenario carefully.

**Sequencing of tumour DNA identifies a somatic alteration that…**	**In this situation, I would…**				
	**Definitely disclose**	**Probably disclose**	**Probably not disclose**	**Definitely not disclose**	**Unsure**
4. Is in a pathway that is *not* targeted by any approved agent. However, an agent that targets this pathway is currently being studied in a phase II clinical trial that's open at your institution. Your patient may be eligible for this trial.	☐	☐	☐	☐	☐
5. Is in a pathway that is targeted by a commercially available agent that is approved *for a different cancer*. There are no reports in the literature of agents that target this pathway being used in your patient's type of cancer.	☐	☐	☐	☐	☐
6. Is known to confer a *favourable* prognosis, compared with the average for patients with this condition. There are no available agents, either commercially or through a clinical trial, that target the relevant pathway.	☐	☐	☐	☐	☐
7. Is known to confer an *unfavourable* prognosis, compared with the average for patients with this condition. There are no available agents, either commercially or through a clinical trial, that target the relevant pathway.	☐	☐	☐	☐	☐

8. For what percentage of your patients do you anticipate that molecularly indicated agents will be available/accessible?

0 50 100

☐☐☐☐☐☐☐☐☐☐☐☐☐☐☐☐☐☐☐☐☐☐☐☐☐

(Please place a mark on the scale above)

Please indicate how confident you are in your ability to do the following things.

	**Very confident**	**Moderately confident**	**A little confident**	**Not confident at all**
9. Ability to interpret somatic (tumour) genomic results in your disease area.	☐	☐	☐	☐
10. Ability to explain somatic genomic concepts to patients.	☐	☐	☐	☐
11. Ability to make treatment recommendations based on somatic genomic information.	☐	☐	☐	☐
12. Ability to identify consultants who have special expertise in integrating somatic genomic information into patients’ care.	☐	☐	☐	☐
13. Ability to provide psychosocial support related to coping with a somatic alteration that has adverse prognostic implications.	☐	☐	☐	☐

The next set of questions is about your use of genomics in practice.

On average, how many times a year do you order or interpret the following types of genetic or genomic tests in your clinical practice?

Include both cases in which you order the test yourself and cases in which you use or interpret the results of tests ordered by others.

	**Approximate Number of Times Per Year**
14. Somatic tests to evaluate for alterations in tumour DNA.	___
15. Germline tests to evaluate for inherited cancer predisposition syndromes.	___
16. Germline tests to evaluate for pharmacogenetic polymorphisms (i.e., that affect drug metabolism or toxicity) related to *cancer drugs*.	___
17. Germline tests to evaluate for pharmacogenetic polymorphisms related to *non-cancer drugs*.	___

18. To what extent does tumour pathology (e.g. histology, immunohistochemistry) inform treatment choices compared with the tumour molecular profile (e.g. EGFR/BRAF)?

Tumour Pathology ________________________________ Tumour Molecular Profile

(Please place a mark on the scale above)

19. Which of the following would be beneficial when receiving a molecular profile report? (Tick all that apply)

- ☐ Genetics Staff (e.g. Clinical Geneticist/Genetic Counsellor) in clinic
- ☐ Genetics Staff on call
- ☐ Genetics Staff available by telephone
- ☐ Genetics Pathologist available by telephone
- ☐ Other____________________________

The final set of questions is about you.

20. How many years has it been since you graduated from medical school?

- ☐ 1-10 years
- ☐ 11-20 years
- ☐ 21-30 years
- ☐ 31-40 years
- ☐ >40 years

21. What is your specialty?

- ☐ Oncology (including Medical/Surgical/Radiation)
- ☐ Respiratory Physician
- ☐
- ☐ Haematologist

Other____________________________

22. On average, how many unique patients do you see for treatment or evaluation each month? Please include both new and established patients. *Your best estimate is fine*.

_____________ number

Do you have any additional thoughts that you wish to share about the issues raised in this survey? Please feel free to write in the space below as we welcome your feedback.

Other

Thank you very much for completing this survey! Your participation is greatly appreciated.

Table S1. Additional qualitative comments shared relating to the survey instrument

Of limited relevance to a general surgeon; it's dealt with by our oncologists
There is also a general lack of knowledge around costs - to the patient and to the system
Some mutations have high reliability in assessment. Whole exome sequencing and many mutations lack testing for in the laboratory, reliability, and therefore their use in clinical practice should be considered unproven until reliability studies are undertaken.
I suspect in the near future - genomics probably will become part and parcel of management of patients with a new diagnosis of cancer
Major issue with sequencing is the lack of infrastructure to counsel the patients. Testing is the smallest component. Until this is in place, you will not see wide roll out.
As a respiratory physician, the main priorities have been - 1. consenting the patient for WES pre-biopsy 2. timely result notification
There is a need for increased and ongoing education of doctors in this aspect of medicine, which is a really exciting area which has already led to far-reaching improvements in cancer therapy.
These tests are being done without thought as to how to appropriately incorporate them into solid tumours. It is currently a wasted resource.
Emerging technology and application; ongoing education is very important.
Currently the way this service is run patients are not selected carefully enough for genomic testing. In the vast majority of cases, it is unhelpful and not a good use of resources. Also, there is no established, smooth way of getting the results to the clinicians who can interpret it. The system needs to be improved.
Much depends on who is funding testing - not appropriate for public fees to be spent on unproven technologies, but I don't have an issue with patients funding tests.
While not a genetics expert, it would greatly assist close consultation and genetic pathologist in our cancer diagnostics - either privately or publicly
Be good to get review/input; we have a genetics counsellor working with us.
There is a lack of genetic counselling/advice available in my area of far North Queensland. Would like to see more of it. QH service overwhelmed by need.
There is emerging data on the non-coding genome such as the promoter and effector regions which are making the science more complicated but also more fascinating in terms of gene experience/effect. So, I do not feel that at a clinic level we are ready to use most of the information practically.
I don't request many WGS studies i.e. foundation one etc.

All answers pertain to the diagnosed cancer related findings not incidental findings potentially related to other disorders
Approaching retirement so not representative of a busy oncology practice.
Need more clinical relevance to mutation; need more trial options
Heavily rely on medical oncology colleagues to order and interpret somatic genomic tests on our breast cancer patients.
Difficult keeping up with rapid development/change in this field

TUMOR SPECIFIC REPORT

Australian Translational Genomics Centre
Queensland University of Technology

**Patient Name**	XXXXXXXXXX	**UR Number**	XXXXXXXXXX
**DOB**	14 Mar 2019	**AusLab Number**	XXXXXXXXXX
**Sex**	XXXXXXXXXX	**FIN**	XXXXXXXXXX

Whole Exome Somatic Test Report

**Report Status:**	FINAL	**Report Type:**	Breast
**Sample Recieve Date:**	14 Mar 2019	**Report Generation Date:**	11 Feb 2019
**Specimen**	XXXXXXXXXX	**Tissue**	XXXXXXXXXX
**QC Status:**	**PASS**		

Samples received all met initial input quality requirements, and have undergone whole exome sequencing and somatic mutations have been identified to assist with the genetic profiling of the tumour (see Methodology section for further details). No second, independent, sample was received. For further information, or questions, about this report, please contact ATGC directly using the contact details at the bottom of this page.

**Tumour Burden:**	5.2 Mutations/Mbp
**Tumour Purity Estimate:**	16-36%

Somatic Mutations Summary

There was 1 reportable variant found in this sample.

Gene	Mutation	Consequence	Variant Allele Frequency
PIK3CA	NP_006209.2:p.Asn345Lys	missense_variant	20.0%

Somatic CNV Summary

There were 3 reportable CNVs found in this sample.

Gene	CNV Type	Copy Number	Start	Stop	Length	Whole/Partial
FGF3	GAIN	9	69,514,027	69,633,701	119,675	Whole
RSF1	GAIN	5	77,402,202	77,475,734	73,533	Partial
RSF1	GAIN	5	77,531,572	77,553,674	22,103	Partial

Somatic Mutations Details

PIK3CA

Gene PIK3CA (missense_variant, VAF = 20.0% Tumour AD = 401,100 Normal AD = 518,2)
HGVSg NC_000003.11:g.178921553T>A
HGVSc NM_006218.2:c.1035T>A
HGVSp NP_006209.2:p.Asn345Lys
COSMIC COSM754 (count 108) FATHMM PATHOGENIC (0.955)

Australian Translational Genomics Centre
Queensland University of Technology

**Patient Name**	XXXXXXXXXX	**UR Number**	XXXXXXXXXX
**DOB**	14 Mar 2019	**AusLab Number**	XXXXXXXXXX
**Sex**	XXXXXXXXXX	**FIN**	XXXXXXXXXX

This mutation is associated with 1 clinical variant

Variant MUTATION, 311 , Transcript Variant, Gain Of Function Variant

Description PIK3CA mutations are observed in many cancers among the most prevalent are breast cancer, colorectal cancer, and head and neck squamous cell carcinoma. These mutations have been observed in up to 40%, 20%, and 21% respectively for each of these cancer types with mutations commonly occurring within the helical exon 9 and kinase domain of exon 20. In breast cancer PIK3CA mutations have been associated with better prognosis, and a greater survival outcome, however the PI3K pathway is also associated with increased drug resistance (trastuzumab /lapatinib). PIK3CA mutations in both HNSCC and CRC have been associated with resistance to anti-EGFR therapeutic agents and mutations in exon 9 and 20 are associated with a poorer prognosis in CRC. Interestingly PIK3CA mutations in HNSCC have been observed to be absent from a number of ethnic groups including greek, german, and vietnamese populations.

This variant has 3 evidence items associated with it

Level	ID	Disease	Type	Direction	Significance	PubMed ID
B	1296	Her2-receptor Positive Breast Cancer	Predictive	Supports	Sensitivity/Response	27091708
B	1384	Her2-receptor Positive Breast Cancer	Predictive	Supports	Resistance	17936563
B	6188	Breast Cancer	Prognostic	Supports	Better Outcome	17575221

Somatic CNV Details

FGF3 - GAIN - Copy number 9

Variant AMPLIFICATION, 630 , Transcript Amplification

Description

This variant has 1 evidence item associated with it

Level	ID	Disease	Type	Direction	Significance	PubMed ID
B	1606	Breast Cancer	Predictive	Supports	Sensitivity/Response	23658459

RSF1 - GAIN - Copy number 5

Variant AMPLIFICATION, 358 , Transcript Amplification

Description

This variant has 1 evidence item associated with it

Level	ID	Disease	Type	Direction	Significance	PubMed ID
B	857	Breast Cancer	Predictive	Supports	Resistance	24367492

RSF1 - GAIN - Copy number 5

**Patient Name**	XXXXXXXXXX	**UR Number**	XXXXXXXXXX
**DOB**	14 Mar 2019	**AusLab Number**	XXXXXXXXXX
**Sex**	XXXXXXXXXX	**FIN**	XXXXXXXXXX

Variant Description AMPLIFICATION, 358 , Transcript Amplification

This variant has 1 evidence item associated with it

Level	ID	Disease	Type	Direction	Significance	PubMed ID
B	857	Breast Cancer	Predictive	Supports	Resistance	24367492

Additional Comments

There are no additional comments for this report

Methodology

DNA libraries were prepared from the received samples and target regions captured using the IDT exome capture kit plus IDT custom spike in. Massively parallel sequencing was performed using the Illumina NextSeq 500 platform to an average depth of 507X for this sample. Data were aligned to the GRCh37 Human Genome reference sequence using the ATGC Bioinformatics Pipeline version and annotated according to the ATGC Clinical Variant Database version.

The assay reports somatic single nucleotide variants (SNV) and small (<100bp) insertions and deletions (INDELs) with a variant allele frequency >3% and the frequency is greater that the percentage of contamination detected plus 1.5%. Copy number variants (CNVs) spanning partial or whole genes are reported when amplification exceeds 5 copies or more and deletions are reported when the tumour purity (proportion of the sample that contains the somatic variant) is estimated to be greater than 60%. Previous validation assays found that the sensitivity of >99.5% for SNVs, >95% for INDELs with VAF >5% and sensitivity to reportable CNVs is >75% for tumour purities >15%. For higher tumour purities where VAF >25%, the sensitivity rises to >99.5% for SNVs and >95% for INDELs. For reportable CNVs sensitivity is >85% when tumour purity is >75% and >60% when tumour purity is >60%. The total mutational burden is a count of SNVs and INDELs within the IDT custom spike in regions predicted to cause protein coding alterations divided by the total number of bases tested (in Mbp).

The following genes were selected for filtering based on documented association with this cancer/tissue type:

ERBB2, FGF3, FGFR1, FGFR2, NCOA3, PIK3CA, PTEN, RSF1, TP53

These genes appear in the ATGC Clinical Variant database and either the genes and/or the specific variants are associated with A or B level evidence.

> **Level A** Validated association: Proven/consensus association in human medicine.
> **Level B** Clinical evidence: Clinical trial or other primary patient data supports association.

In some instances, all somatic protein coding variants within a specific gene are deemed reportable. In order to ascertain whether a variant affects protein coding the Variant Effect Predictor categorises the variants using RefSeq transcripts and results are reported using HGVS nomenclature (https://varnomen.hgvs.org).

Rare errors in annotation can arise due to the presence of genomic transcripts not described in RefSeq. Alternatively, multiple genomic variations may combine to produce undetected protein coding changes. This could generate false positive and false negative results. The assay is not designed to ascertain whether the identified variants alter expression in the tissue sample.

Australian Translational Genomics Centre
Queensland University of Technology

**Patient Name**	XXXXXXXXXX	**UR Number**	XXXXXXXXXX
**DOB**	14 Mar 2019	**AusLab Number**	XXXXXXXXXX
**Sex**	XXXXXXXXXX	**FIN**	XXXXXXXXXX

For **research use only** information about mutations outside of the genes listed above, please contact ATGC directly via the contact details at the bottom of this page.

**Tumour ID:**	20170724-0003	**Workflow Version:**	3.2.0
**Normal ID:**	20170724-0001	**Workflow Commit:**	e42932266e4a8d4beb3df16c6877b07a7e311b5b
**Clinical DB Version:**	2018-11-27.2	**Report Software Version:**	1.4.0
**VEP Version:**	v91	**Report Software Commit:**	444d1a491c89898ac4e6dad28e908861e7e0828a
**Run ID:**	171005_NB501489_0080_AHL2Y5BGX2	**Analysis ID:**	f408369d-d93d-4c49-93a1-eb304d9bd62a

Disclaimer

The methods used to produce this report have been validated by Australian Translational Genomics Centre (ATGC) at Queensland University of Technology. Nonetheless, there is a chance that false positive and false negative results may occur as Pathology Queensland and ATGC are not in control of the tissue collection. Tissue collection factors that may give rise to false positive or false negative results include tissue heterogeneity, insufficient tissue quality, or contamination. A list of methodology including the bioinformatics pipeline utilised to draft the report are noted above. Diseases are influenced by many factors, including epigenetic and environmental variables that may not be addressed by this report, and, as such, the report should not be interpreted in isolation. Any diagnosis, or prognosis, should consider all pertinent clinical information in addition to this report. The bioinformatics pipeline used to identify the variants reported has also been validated by ATGC. The analysis limitations are outlined in the methodology section of this report.

The clinical significance of many variants is not well understood and interpretation of variants may change over time. Interpretation of variants in this report is performed to the best knowledge of the laboratory based on the information available at the time of reporting. Re-analysis of variants in previously issued reports in light of new evidence is not routinely performed, but may be available upon request

PAN-CANCER REPORT

Australian Translational Genomics Centre
Queensland University of Technology

**Patient Name**	XXXXXXXXXX	**UR Number**	XXXXXXXXXX
**DOB**	14 Mar 2019	**AusLab Number**	XXXXXXXXXX
**Sex**	XXXXXXXXXX	**FIN**	XXXXXXXXXX

Whole Exome Somatic Test Report

THIS REPORT IS FOR RESEARCH USE ONLY

**Report Status:**	FINAL	**Report Type:**	Pan
**Sample Recieve Date:**	14 Mar 2019	**Report Generation Date:**	11 Feb 2019
**Specimen**	XXXXXXXXXX	**Tissue**	XXXXXXXXXX
**QC Status:**	**PASS**		

Samples received all met initial input quality requirements, and have undergone whole exome sequencing and somatic mutations have been identified to assist with the genetic profiling of the tumour (see Methodology section for further details). No second, independent, sample was received. For further information, or questions, about this report, please contact ATGC directly using the contact details at the bottom of this page.

**Tumour Burden:**	5.2 Mutations/Mbp
**Tumour Purity Estimate:**	16-36%

Somatic Mutations Summary

There were 2 reportable variants found in this sample.

Gene	Mutation	Consequence	Variant Allele Frequency
PIK3CA	NP_006209.2:p.Asn345Lys	missense_variant	20.0%
FANCC	NP_001230672.1:p.Ala547Val	missense_variant	17.9%

Somatic CNV Summary

There were 10 reportable CNVs found in this sample.

Gene	CNV Type	Copy Number	Start	Stop	Length	Whole/Partial
MSH2	LOSS	0	47,596,643	47,748,169	151,527	Whole
MSH6	LOSS	0	47,797,458	48,011,377	213,920	Partial
MSH6	LOSS	0	48,023,031	48,066,813	43,783	Partial
FANCC	LOSS	0	97,887,366	98,011,573	124,208	Partial
CCND1	GAIN	5	69,456,080	69,490,007	33,928	Whole
FGF3	GAIN	9	69,514,027	69,633,701	119,675	Whole
RSF1	GAIN	5	77,402,202	77,475,734	73,533	Partial
RSF1	GAIN	5	77,531,572	77,553,674	22,103	Partial
NF2	LOSS	0	29,438,481	30,138,469	699,989	Whole

**Patient Name**	XXXXXXXXXX	**UR Number**	XXXXXXXXXX
**DOB**	14 Mar 2019	**AusLab Number**	XXXXXXXXXX
**Sex**	XXXXXXXXXX	**FIN**	XXXXXXXXXX

SOX10	LOSS	0	38,307,957	38,379,808	71,852	Whole

Somatic Mutations Details

PIK3CA

**Gene**	PIK3CA (missense_variant, VAF = 20.0% Tumour AD = 401,100 Normal AD = 518,2)
**HGVSg**	NC_000003.11:g.178921553T>A
**HGVSc**	NM_006218.2:c.1035T>A
**HGVSp**	NP_006209.2:p.Asn345Lys
**COSMIC**	COSM754 (count 108) FATHMM PATHOGENIC (0.955)

This mutation is associated with 1 clinical variant

**Variant**	MUTATION, 311 , Transcript Variant, Gain Of Function Variant
**Description**	PIK3CA mutations are observed in many cancers among the most prevalent are breast cancer, colorectal cancer, and head and neck squamous cell carcinoma. These mutations have been observed in up to 40%, 20%, and 21% respectively for each of these cancer types with mutations commonly occurring within the helical exon 9 and kinase domain of exon 20. In breast cancer PIK3CA mutations have been associated with better prognosis, and a greater survival outcome, however the PI3K pathway is also associated with increased drug resistance (trastuzumab /lapatinib). PIK3CA mutations in both HNSCC and CRC have been associated with resistance to anti-EGFR therapeutic agents and mutations in exon 9 and 20 are associated with a poorer prognosis in CRC. Interestingly PIK3CA mutations in HNSCC have been observed to be absent from a number of ethnic groups including greek, german, and vietnamese populations.

This variant has 20 evidence items associated with it

Australian Translational Genomics Centre
Queensland University of Technology

**Patient Name**	XXXXXXXXXX	**UR Number**	XXXXXXXXXX
**DOB**	14 Mar 2019	**AusLab Number**	XXXXXXXXXX
**Sex**	XXXXXXXXXX	**FIN**	XXXXXXXXXX

Level	ID	Disease	Type	Direction	Significance	PubMed ID
B	915	Colorectal Cancer	Predictive	Supports	Resistance	23435830
B	1296	Her2-receptor Positive Breast Cancer	Predictive	Supports	Sensitivity/Response	27091708
B	1384	Her2-receptor Positive Breast Cancer	Predictive	Supports	Resistance	17936563
B	3040	Cancer	Predictive	Supports	Sensitivity/Response	28489509
B	6188	Breast Cancer	Prognostic	Supports	Better Outcome	17575221
B	6301	Colorectal Cancer	Predictive	Supports	Resistance	19603024
B	6362	Colorectal Cancer	Predictive	Supports	Resistance	19223544
B	6375	Colorectal Cancer	Predictive	Supports	Sensitivity/Response	23094721
D	771	Colorectal Cancer	Predictive	Supports	Sensitivity/Response	25242168
D	1360	Head And Neck Cancer	Predictive	Supports	Sensitivity/Response	23619167
D	1402	Cancer	Predictive	Supports	Sensitivity/Response	24608574
D	1490	Head And Neck Squamous Cell Carcinoma	Predictive	Supports	Sensitivity/Response	26589432
D	1501	Stomach Cancer	Predictive	Supports	Sensitivity/Response	24088382
D	1503	Cancer	Predictive	Supports	Sensitivity/Response	22294718
D	1504	Her2-receptor Positive Breast Cancer	Predictive	Supports	Sensitivity/Response	22294718
D	1600	Breast Cancer	Predictive	Supports	Sensitivity/Response	25002028
D	1607	Breast Cancer	Predictive	Supports	Sensitivity/Response	25002028
D	1610	Breast Cancer	Predictive	Supports	Sensitivity/Response	21358673
D	1616	Endometrial Cancer	Predictive	Supports	Sensitivity/Response	23674493
D	1705	Colorectal Cancer	Predictive	Supports	Resistance	22586653

FANCC

Gene FANCC (missense_variant, VAF = 17.9% Tumour AD = 102,22 Normal AD = 140,1)
HGVSg NC_000009.11:g.97864026G>A
HGVSc NM_001243743.1:c.1640C>T
HGVSp NP_001230672.1:p.Ala547Val
COSMIC This mutation does not appear in COSMIC

This mutation is associated with 1 clinical variant

Variant Description LOSS-OF-FUNCTION, 534 , Loss Of Function Variant

This variant has 1 evidence item associated with it

Level	ID	Disease	Type	Direction	Significance	PubMed ID
D	1307	Pancreatic Cancer	Predictive	Supports	Sensitivity/Response	16243825

ATGC Contact Details
Ph: +61 7 3443 7364
Email: atgpq@qut.edu.au

**Patient Name**	XXXXXXXXXX	**UR Number**	XXXXXXXXXX
**DOB**	14 Mar 2019	**AusLab Number**	XXXXXXXXXX
**Sex**	XXXXXXXXXX	**FIN**	XXXXXXXXXX

Somatic CNV Details

MSH2 - LOSS - Copy number 0

Variant LOSS, 808 , Loss Of Function Variant
Description

This variant has 1 evidence item associated with it

Level	ID	Disease	Type	Direction	Significance	PubMed ID
C	1877	Urothelial Carcinoma	Predictive	Supports	Sensitivity/Response	26674132

MSH6 - LOSS - Copy number 0

Variant LOSS, 809 , Loss Of Function Variant
Description

This variant has 1 evidence item associated with it

Level	ID	Disease	Type	Direction	Significance	PubMed ID
C	1878	Urothelial Carcinoma	Predictive	Supports	Sensitivity/Response	26674132

MSH6 - LOSS - Copy number 0

Variant LOSS, 809 , Loss Of Function Variant
Description

This variant has 1 evidence item associated with it

Level	ID	Disease	Type	Direction	Significance	PubMed ID
C	1878	Urothelial Carcinoma	Predictive	Supports	Sensitivity/Response	26674132

FANCC - LOSS - Copy number 0

Variant LOSS-OF-FUNCTION, 534 , Loss Of Function Variant
Description

This variant has 1 evidence item associated with it

Level	ID	Disease	Type	Direction	Significance	PubMed ID
D	1307	Pancreatic Cancer	Predictive	Supports	Sensitivity/Response	16243825

Australian Translational Genomics Centre
Queensland University of Technology

**Patient Name**	XXXXXXXXXX	**UR Number**	XXXXXXXXXX
**DOB**	14 Mar 2019	**AusLab Number**	XXXXXXXXXX
**Sex**	XXXXXXXXXX	**FIN**	XXXXXXXXXX

CCND1 - GAIN - Copy number 5

Variant AMPLIFICATION, 18 , Transcript Amplification
Description CCND1 amplification has been implicated in poorer prognosis in non-small cell lung cancer.

This variant has 3 evidence items associated with it

Level	ID	Disease	Type	Direction	Significance	PubMed ID
B	354	Non-small Cell Lung Carcinoma	Prognostic	Supports	Poor Outcome	17070615
B	1495	Skin Melanoma	Predictive	Supports	Sensitivity/Response	26307133
D	1562	Breast Cancer	Predictive	Supports	Sensitivity/Response	19874578

FGF3 - GAIN - Copy number 9

Variant AMPLIFICATION, 630 , Transcript Amplification
Description

This variant has 1 evidence item associated with it

Level	ID	Disease	Type	Direction	Significance	PubMed ID
B	1606	Breast Cancer	Predictive	Supports	Sensitivity/Response	23658459

RSF1 - GAIN - Copy number 5

Variant AMPLIFICATION, 358 , Transcript Amplification
Description

This variant has 1 evidence item associated with it

Level	ID	Disease	Type	Direction	Significance	PubMed ID
B	857	Breast Cancer	Predictive	Supports	Resistance	24367492

RSF1 - GAIN - Copy number 5

Variant AMPLIFICATION, 358 , Transcript Amplification
Description

This variant has 1 evidence item associated with it

Level	ID	Disease	Type	Direction	Significance	PubMed ID
B	857	Breast Cancer	Predictive	Supports	Resistance	24367492

**Patient Name**	XXXXXXXXXX	**UR Number**	XXXXXXXXXX
**DOB**	14 Mar 2019	**AusLab Number**	XXXXXXXXXX
**Sex**	XXXXXXXXXX	**FIN**	XXXXXXXXXX

NF2 - LOSS - Copy number 0

Variant LOSS, 697 , Loss Of Function Variant
Description

This variant has 1 evidence item associated with it

Level	ID	Disease	Type	Direction	Significance	PubMed ID
D	1742	Thyroid Carcinoma	Predictive	Supports	Sensitivity/Response	26359368

SOX10 - LOSS - Copy number 0

Variant LOSS, 672 , Loss Of Function Variant
Description

This variant has 1 evidence item associated with it

Level	ID	Disease	Type	Direction	Significance	PubMed ID
D	1710	Melanoma	Predictive	Supports	Resistance	24670642

Additional Comments

The following genes had a proportion of bases with lower than normal coverage. The following lists the percentage of coding bases in tested genes with less than 60X coverage. While mutation may still be called in these regions, the sensitivity may be lower.

ASNS (53.68%), AURKA (40.43%), B2M (64.17%), BIRC3 (54.88%), BTK (70.08%), CALR (43.38%), CCNE1 (27.09%), CDK4 (16.23%), CEBPA (10.49%), CRBN (24.68%), ERBB3 (24.71%), ESR1 (16.73%), FANCC (15.11%), FOXL2 (17.95%), FOXP1 (11.04%), GNA11 (21.67%), GNAQ (40.74%), GNAS (12.15%), HSPH1 (16.62%), JAK2 (23.98%), KDR (15.16%), MAPK1 (48.48%), MDM2 (21.95%), MLH1 (30.82%), MRE11 (34.46%), MSH2 (29.88%), MYCN (30.92%), NF2 (36.57%), NT5C2 (56.47%), POLE (14.52%), POT1 (93.65%), PRDM1 (40.65%), PTCH1 (33.93%), PTPRB (55.66%), RAC1 (82.86%), RAF1 (62.30%), REL (28.82%), RHOA (23.72%), RICTOR (37.08%), RIT1 (15.33%), ROS1 (16.89%), SMARCA4 (31.02%), SMARCB1 (44.39%), SMO (15.86%), SOX10 (12.71%), TOP1 (26.94%), TYMS (17.73%)

Methodology

DNA libraries were prepared from the received samples and target regions captured using the IDT exome capture kit plus IDT custom spike in. Massively parallel sequencing was performed using the Illumina NextSeq 500 platform to an average depth of 507X for this sample. Data were aligned to the GRCh37 Human Genome reference sequence using the ATGC Bioinformatics Pipeline version and annotated according to the ATGC Clinical Variant Database version.

The assay reports somatic single nucleotide variants (SNV) and small (<100bp) insertions and deletions (INDELs) with a variant allele frequency >3% and the frequency is greater that the percentage of contamination detected plus 1.5%. Copy number variants (CNVs) spanning partial or whole genes are reported when amplification exceeds 5 copies or more and deletions are reported when the tumour purity (proportion of the sample that contains the somatic variant) is estimated to be greater than 60%. Previous validation assays found that the sensitivity of >99.5% for SNVs, >95% for INDELs with VAF >5% and sensitivity to reportable CNVs is >75% for tumour purities >15%. For higher tumour purities where VAF >25%, the sensitivity rises to >99.5% for SNVs and >95% for INDELs. For reportable CNVs sensitivity is >85% when tumour purity is >75% and >60% when tumour purity is

**Patient Name**	XXXXXXXXXX	**UR Number**	XXXXXXXXXX
**DOB**	14 Mar 2019	**AusLab Number**	XXXXXXXXXX
**Sex**	XXXXXXXXXX	**FIN**	XXXXXXXXXX

>60%. The total mutational burden is a count of SNVs and INDELs within the IDT custom spike in regions predicted to cause protein coding alterations divided by the total number of bases tested (in Mbp).

The following genes were selected for filtering based on documented association with this cancer/tissue type:

ABCC3, ABL1, ACVR1, AKT1, AKT2, AKT3, ALK, APC, AR, ARAF, ASNS, ASS1, ASXL1, ATM, ATR, AURKA, B2M, BAP1, BCOR, BIRC3, BIRC7, BRAF, BRCA1, BRCA2, BTK, CALR, CCND1, CCND3, CCNE1, CDH1, CDK12, CDK4, CDKN2A, CDKN2B, CEBPA, CRBN, CSF3R, CTNNB1, DDR2, DNMT3A, EGFR, ERBB2, ERBB3, ERBB4, ERRFI1, ESR1, EZH2, FANCC, FBXW7, FGF3, FGFR1, FGFR2, FGFR3, FLT3, FOXL2, FOXP1, GNA11, GNAQ, GNAS, GSTP1, HRAS, HSPH1, IDH1, IDH2, JAK1, JAK2, KDR, KIT, KMT2C, KMT2D, KRAS, LRP1B, MAP2K1, MAPK1, MDM2, MEN1, MET, MLH1, MRE11, MSH2, MSH6, MTOR, MYCN, MYD88, NCOA3, NF2, NOTCH1, NPM1, NRAS, NT5C2, NTRK1, NTRK3, PBRM1, PDGFRA, PIK3CA, PIK3R1, PML, POLE, POT1, PRDM1, PTCH1, PTEN, PTPRB, PTPRD, RAC1, RAF1, RB1, REL, RET, RHOA, RICTOR, RIT1, ROS1, RSF1, RUNX1, SETBP1, SF3B1, SMAD4, SMARCA4, SMARCB1, SMO, SOX10, SRSF2, STAG2, STAT3, STK11, TERT, TET2, TOP1, TP53, TSC1, TSC2, TYMS, U2AF1, VHL, WT1

These genes appear in the ATGC Clinical Variant database and either the genes and/or the specific variants are associated with A, B, C, D or E level evidence.

Level A Validated association: Proven/consensus association in human medicine.
Level B Clinical evidence: Clinical trial or other primary patient data supports association.
Level C Case Study: Individual case reports from clinical journals
Level D Preclinical evidence
Level E Inferential evidence, indirect evidence

In some instances, all somatic protein coding variants within a specific gene are deemed reportable. In order to ascertain whether a variant affects protein coding the Variant Effect Predictor categorises the variants using RefSeq transcripts and results are reported using HGVS nomenclature (https://varnomen.hgvs.org).

Rare errors in annotation can arise due to the presence of genomic transcripts not described in RefSeq. Alternatively, multiple genomic variations may combine to produce undetected protein coding changes. This could generate false positive and false negative results. The assay is not designed to ascertain whether the identified variants alter expression in the tissue sample.

For **research use only** information about mutations outside of the genes listed above, please contact ATGC directly via the contact details at the bottom of this page.

**Tumour ID:**	20170724-0003	**Workflow Version:**	3.2.0
**Normal ID:**	20170724-0001	**Workflow Commit:**	e42932266e4a8d4beb3df16c6877b07a7e311b5b
**Clinical DB Version:**	2018-11-27.2	**Report Software Version:**	1.4.0
**VEP Version:**	v91	**Report Software Commit:**	444d1a491c89898ac4e6dad28e908861e7e0828a
**Run ID:**	171005_NB501489_0080_AHL2Y5BGX2	**Analysis ID:**	f408369d-d93d-4c49-93a1-eb304d9bd62a

Disclaimer

The methods used to produce this report have been validated by Australian Translational Genomics Centre (ATGC) at Queensland University of Technology. Nonetheless, there is a chance that false positive and false negative results may occur as Pathology Queensland and ATGC are not in control of the tissue collection. Tissue collection factors that may give rise to false positive or false negative results include tissue heterogeneity, insufficient tissue quality, or contamination. A list of methodology including the bioinformatics pipeline utilised to draft the report are noted above. Diseases are influenced by many factors, including epigenetic and environmental variables that may not be addressed by this report, and, as such, the report should not be interpreted

**Patient Name**	XXXXXXXXXX	**UR Number**	XXXXXXXXXX
**DOB**	14 Mar 2019	**AusLab Number**	XXXXXXXXXX
**Sex**	XXXXXXXXXX	**FIN**	XXXXXXXXXX

in isolation. Any diagnosis, or prognosis, should consider all pertinent clinical information in addition to this report. The bioinformatics pipeline used to identify the variants reported has also been validated by ATGC. The analysis limitations are outlined in the methodology section of this report.

The clinical significance of many variants is not well understood and interpretation of variants may change over time. Interpretation of variants in this report is performed to the best knowledge of the laboratory based on the information available at the time of reporting. Re-analysis of variants in previously issued reports in light of new evidence is not routinely performed, but may be available upon request
